# Continuous versus discrete robotic feedback for brain-computer interfaces aimed for neurorehabilitation

**DOI:** 10.3389/fnbot.2023.1015464

**Published:** 2023-02-28

**Authors:** Ruben I. Carino-Escobar, Martín E. Rodríguez-García, Paul Carrillo-Mora, Raquel Valdés-Cristerna, Jessica Cantillo-Negrete

**Affiliations:** ^1^Division of Research in Medical Engineering, Instituto Nacional de Rehabilitación Luis Guillermo Ibarra Ibarra, Mexico City, Mexico; ^2^Electrical Engineering Department, Universidad Autónoma Metropolitana Unidad Iztapalapa, Mexico City, Mexico; ^3^Division of Neuroscience, Instituto Nacional de Rehabilitación Luis Guillermo Ibarra Ibarra, Mexico City, Mexico

**Keywords:** EEG, BCI, BMI, neurofeedback, motor imagery, robot

## Abstract

**Introduction:**

Brain-Computer Interfaces (BCI) can allow control of external devices using motor imagery (MI) decoded from electroencephalography (EEG). Although BCI have a wide range of applications including neurorehabilitation, the low spatial resolution of EEG, coupled to the variability of cortical activations during MI, make control of BCI based on EEG a challenging task.

**Methods:**

An assessment of BCI control with different feedback timing strategies was performed. Two different feedback timing strategies were compared, comprised by passive hand movement provided by a robotic hand orthosis. One of the timing strategies, the continuous, involved the partial movement of the robot immediately after the recognition of each time segment in which hand MI was performed. The other feedback, the discrete, was comprised by the entire movement of the robot after the processing of the complete MI period. Eighteen healthy participants performed two sessions of BCI training and testing, one with each feedback.

**Results:**

Significantly higher BCI performance (65.4 ± 17.9% with the continuous and 62.1 ± 18.6% with the discrete feedback) and pronounced bilateral alpha and ipsilateral beta cortical activations were observed with the continuous feedback.

**Discussion:**

It was hypothesized that these effects, although heterogenous across participants, were caused by the enhancement of attentional and closed-loop somatosensory processes. This is important, since a continuous feedback timing could increase the number of BCI users that can control a MI-based system or enhance cortical activations associated with neuroplasticity, important for neurorehabilitation applications.

## 1. Introduction

Brain-Computer Interfaces (BCI) are systems that allow control of external devices using information extracted from the central nervous system (Wolpaw et al., 2002). The most common type of non-invasive BCI designs decode users’ intentions from their electroencephalogram (EEG). In order for users to encode their intentions in the EEG, different strategies or paradigms have been proposed, such as P300, steady-state visual evoked potentials, and motor imagery (MI) (Rashid et al., 2020). Particularly, MI, the mental rehearsal of the movement of a limb such as the hand or foot, can elicit cortical activations similar to those observed during passive and actual movement (Kraeutner et al., 2014). An advantage of the MI paradigm is that it is endogenous (BCI users do not need an external stimulus for controlling the system). Therefore, several applications of MI-based BCI have been proposed, ranging from entertainment to neurorehabilitation (Plass-Oude Bos et al., 2010; Monge-Pereira et al., 2017). However, the non-stationary nature of the EEG signal, its low spatial resolution, and the variability of cortical activations during the performance of MI, makes the correct identification of users’ intentions with this paradigm a challenging task (Rashid et al., 2020).

Different strategies have been reported for increasing MI-based BCI control. For example, feature extraction and classification algorithms that comprise the BCI processing stage can affect user control with the system, since these algorithms allow to identify individuals’ EEG patterns of MI. For this reason, extensive research has been performed regarding feature extraction and classification algorithms in MI-based BCI applications (Blankertz et al., 2008; Ang et al., 2012; Carino-Escobar et al., 2018; Tang et al., 2019). The feedback stage of a BCI, which indicates the degree of performance that users had with the system, can also impact the ability to control the BCI. Specifically, MI-based BCI feedbacks can be mainly cataloged into visual, such as the simple movement of a cursor or the realistic movement of the imagined limb displayed in a virtual reality setup (Choi et al., 2020), and kinesthetic feedbacks, like vibration or passive movement exerted by a robotic device (Cantillo-Negrete et al., 2019; Fleury et al., 2020). It has been reported that kinesthetic feedbacks allow users to elicit more pronounced cortical activations compared to other feedback modalities (Shu et al., 2017; Cantillo-Negrete et al., 2019). This is positive for BCI control since enhanced cortical activations permit processing stages to better differentiate between MI tasks. Enhanced cortical activations can also be desirable for neurorehabilitation applications because they have been associated with neuroplasticity, a recovery mechanism of neurological diseases such as stroke (Ward and Cohen, 2004; Pekna et al., 2012).

Some studies have reported EEG-based BCI performance and cortical activity changes with different types of feedback. Vourvopoulos et al. (2016) compared different types of visual feedback, reporting a lack of performance difference between a virtual reality feedback and a simple 2-D feedback, albeit more pronounced cortical activations were observed with the virtual reality feedback. Barsotti et al. (2018) reported higher BCI performance and more stable cortical activity with a vibrotactile feedback compared with a solely visual feedback. Cantillo-Negrete et al. (2019) reported higher BCI performance and cortical activations with a kinesthetic robotic feedback compared to a visual feedback. However, the timing of these BCI feedbacks within the MI paradigm might also affect performance and elicited cortical activity. This has been remarked by Fleury et al. (2020) by defining two possible types of feedback timing, discrete and continuous. A discrete feedback is presented to the BCI user at the end of a MI trial, after the processing of all the EEG time windows in which MI was performed. On the other hand, a continuous feedback is presented to the BCI user immediately after one of the trial’s MI time windows is processed, and thus, can be presented multiple times during a single trial. Shu et al. (2017) reported one of the few studies regarding the effects of a continuous kinesthetic feedback in a MI-based BCI, by assessing vibrotactile stimulation effects in BCI control and cortical activity when provided at the same time as hand MI was performed. However, to the authors’ knowledge, a direct comparison between a same feedback’s continuous and discrete timing effects in BCI performance and cortical activations has not been reported. This is important, since one type of feedback timing could increase the number of BCI users that can achieve control of a MI system or could enhance cortical activations associated with neuroplasticity, which is important for neurorehabilitation applications.

In this study, a comparison between a continuous and a discrete kinesthetic feedback is presented in a MI-based BCI application. The kinesthetic feedback was comprised by passive hand movement elicited by a robotic hand orthosis. It was hypothesized that the continuous feedback would elicit more pronounced cortical activations in the somatosensory cortex during a hand MI task, improving BCI control. The manuscript is organized as follows: the sample, the BCI system, both feedback types, the experimental procedure, the BCI metrics, and the statistical analysis are detailed. This is followed by results regarding a comparison between BCI metrics with the continuous and discrete feedbacks and by a discussion of these results. Finally, conclusions regarding the effects of both feedbacks on BCI control and cortical activations are presented.

## 2. Materials and methods

### 2.1. Participants

Eighteen healthy participants were included in this study, nine females and nine males, aged between 22 and 32 years (mean 25.3 ± 3). All participants were right-handed according to the Edinburgh Inventory for handedness (Oldfield, 1971), naïve to BCI control, had normal or corrected-to-normal vision, and did not report neurological pathologies or lesions. The research was conducted as part of a study approved by the Ethical and Research Committees of the National Institute of Rehabilitation “Luis Guillermo Ibarra Ibarra” (registry number 25/19AC). An informed consent was signed by all participants.

### 2.2. Brain-computer interface system

The BCI system was comprised by an EEG acquisition stage, a computer monitor to provide instructions to the participants, a processing stage, and the feedback stage. EEG recordings were performed at a sampling rate of 256 Hz with a g.USBamp biosignal amplifier (g.tec medical engineering GmbH, Schiedlberg, Austria) and 16 g.LADYBIRD active electrodes from the same manufacturer. These electrodes were located in the F3, FC3, C5, C3, C1, CP3, P3, FCz, Cz, F4, FC4, C6, C4, C2, CP4, and P4 positions of the international 10–20 system. The reference and ground electrodes were placed in the right earlobe and in the AFz position, respectively. Impedances were kept below 5 kΩ for all recordings.

The processing stage was implemented in a Precision 5820 workstation (Dell Inc., Texas, USA) through a Graphical User Interface programmed in MATLAB (MathWorks Inc., Massachusetts, USA). It was comprised by a calibration (offline) and a testing (online) mode. In the calibration mode, pre-recorded EEG data were filtered in the bands 8–12, 12–16, 16–20, 20–24, 24–28, and 28–32 Hz with 30th order band-pass and notch FIR filters. Afterward, logarithmic variance features for recognizing between tasks were extracted using Common Spatial Patterns (CSP) from 1-s windows of each trial, channel, and frequency band, encompassing the Filter Bank Common Spatial Patterns algorithm (Blankertz et al., 2008; Ang et al., 2012). CSP relevant features, selected with Particle Swarm Optimization (PSO), were used to train a Linear Discriminant Analysis (LDA) classifier (Shi and Eberhart, 1998). Thus, the calibration mode produced subject-specific frequency bands, spatial filters, and LDA coefficients that were used in the testing mode. In the testing mode, 1-s windows of EEG data were acquired, temporal and notch filtered in the frequency bands selected with PSO, and classified into two tasks, using the computed LDA and CSP parameters. Further details of the BCI system processing stage can be found in the work of Cantillo-Negrete et al. (2018).

**FIGURE 1:**
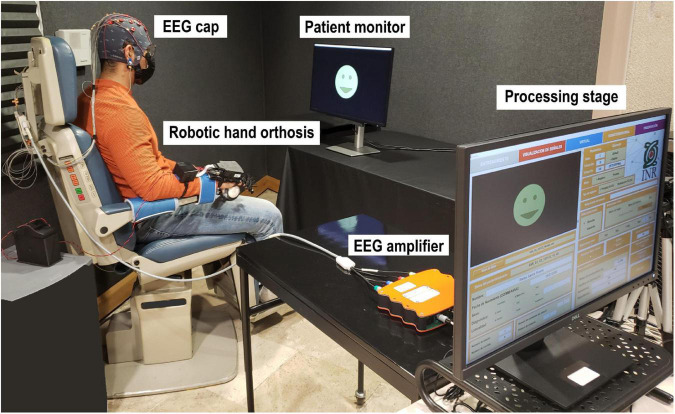
Brain-computer interface setup employed for the experiment. A participant is shown while attempting to control the robotic hand orthosis using motor imagery.

The system had a kinesthetic feedback stage comprised by a robotic orthosis that was placed in the participants’ right hand. This robotic orthosis could elicit passive finger flexion and extension, using a screw mechanism in each finger that converted rotational motion, provided by an electric motor, into linear motion. The orthosis was manufactured for the study, using 3D-printing technology, and aimed at BCI applications. The maximum linear displacement that could be exerted by the orthosis was 5.5 cm in either flexion or extension movements, which was translated into approximately 70–85% of fingers’ maximum movement range. The orthosis velocity was approximately 1.4 cm/s, measured as the time that was needed to complete a full extension of the finger mechanisms in the absence of a load. The orthosis and its circuitry weighted 386 g and measured 23 and 14 cm in length and width, respectively. The orthosis was activated using a Bluetooth communication command transmitted *via* the workstation, generated as an output by the BCI processing stage. Figure 1 depicts the BCI system with each of its stages.

### 2.3. Continuous and discrete feedbacks

**FIGURE 2:**
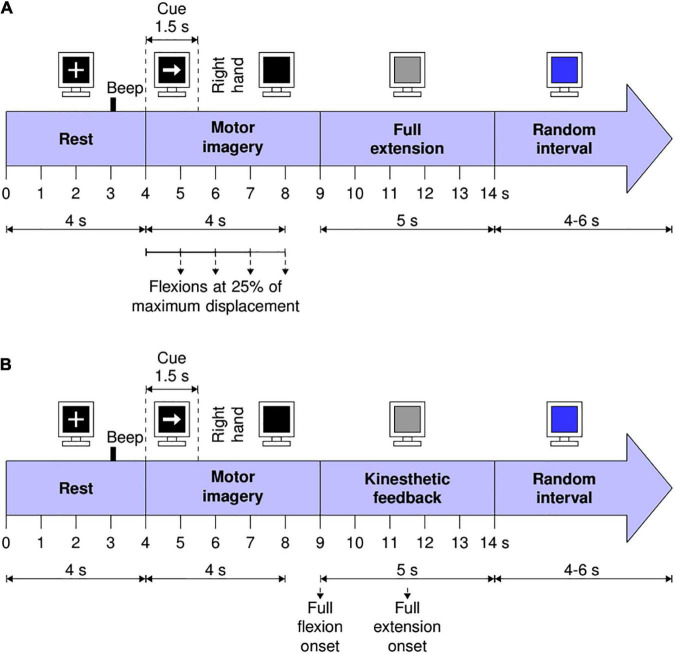
Visual instructions and time structure of the trials with: **(A)** The continuous feedback and **(B)** The discrete feedback.

Continuous feedback trials were comprised by the time structure shown in Figure 2A. First, a rest period of 4 s was presented in which the participant observed a white cross on the computer screen. Three seconds after initiating the rest period a beep sound was played by the monitor’s speakers, alerting the participant of the incoming MI task. Four seconds after the trial’s onset, a white arrow pointing to the right side of the monitor appeared on the computer screen, indicating the participant to start performing right-hand MI (the instruction was: imagine that you grasp and release the baseball placed under the palm of your right hand). This arrow lasted 1.5 s and afterward disappeared, turning the computer screen black for another 3.5 s. Participants were instructed to continue to perform right-hand MI while the black screen was on. Immediately after the BCI system classified a 1-s window from the 4 to 8 s of the trial’s time structure (the MI period) as right-hand MI, participants received passive finger flexion at 25% of the orthosis’ maximum displacement (i.e., a 1.38 cm movement). Hence, a maximum of four flexion movements could be elicited per trial. At 9 s from the trial’s onset, the orthosis performed finger extension movement if the participant previously received finger flexion. Regardless of the orthosis activation, the screen turned gray for 5 s after 9 s from the trial onset, indicating the participant to stop performing the MI task. Finally, the computer screen turned blue after 14 s from the trials’ onset, in which participants were allowed to move, blink, and relax. This blue screen lasted randomly between 4 and 6 s to prevent habituation.

The time structure of trials with the discrete feedback is shown in Figure 2B. The time structure was the same as for the continuous feedback. However, for trials with the discrete feedback, if three or more of the 1-s windows processed by the BCI during the MI task (within the range of 4–8 s of the trial’s structure) were classified as MI, the robotic orthosis performed a single flexion movement until its maximum displacement (5.5 cm) was reached, followed by an extension movement to return to its original position. This complete movement sequence initialized at 9 s from the trials’ onset. The structure of trials with each feedback was based on the Graz paradigm and on previous studies that allowed to infer that the proposed time structures were feasible from the computational and user perspective (Cantillo-Negrete et al., 2018, 2021).

### 2.4. Experimental procedure

All recordings took place in a sound-attenuated environment to reduce external perturbations. Participants were instructed to seat in a comfortable armchair with a computer monitor placed in front of them at approximately 1.5 m. Recordings were done across two sessions in two consecutive days; participants received either the continuous or the discrete feedback in each session. The sequence in which each participant received first a continuous or a discrete session was randomized.

Each session was comprised by a calibration and a testing phase. In each phase, participants were instructed to perform a total of 80 trials of right-hand MI, distributed in four runs of 20 trials each with at least 1 min of rest between runs. During the calibration phase, EEG information was recorded and analyzed to set the BCI subject-specific parameters for the testing phase (as described in the BCI System section). In this phase, participants always received the corresponding feedback in each trial regardless of the BCI system processing stage’s outputs (Cantillo-Negrete et al., 2018). In the testing phase, participants received the corresponding feedback only if the BCI system determined that the MI task was correctly performed.

### 2.5. Brain-computer interface system performance

The percentages of sensitivity (Sens) and classification accuracy (CA) were used to measure participants’ performance during the testing phase of each session. Sens reflects the participants’ ability to control the robotic orthosis during the execution of the MI task, whereas CA assesses participants’ overall performance with the BCI system (ability to elicit MI when instructed and to not elicit it during the rest period). Sens and CA were computed for each participant using the following definitions:

• True Positives: number of 1-s windows correctly classified by the BCI system as MI in a trial during the execution of the MI task.
• True Negatives: number of 1-s windows correctly determined as rest by the BCI system in a trial during the rest period.
• False Positives: number of 1-s windows wrongly detected as MI during the rest period.
• False Negatives: number of 1-s windows wrongly classified as rest during the execution of the MI task.

### 2.6. Electroencephalogram analysis

Due to the modulation of sensorimotor rhythms during hand MI, alpha (8–13 Hz) and beta (14–30 Hz) frequency bands were analyzed (Pfurtscheller et al., 2006; Leuthardt et al., 2009). Recorded raw EEG data from each trial of the testing phase was processed with a 30th order FIR filter from 8 to 30 Hz, and a common average reference (CAR) spatial filter to reduce reference placement effects in EEG recordings (Bertrand et al., 1985). Afterward, a visual inspection of EEG channel data was performed to eliminate trials with excessive noise artifacts. Cortical activations were quantified using event-related desynchronization/synchronization (ERD/ERS) (Pfurtscheller et al., 1999). To this end, power was calculated for each participant and trial in the alpha and beta frequency bands using a time-frequency wavelet analysis (Tallon-Baudry et al., 1997). Complex Morlet wavelets (CMW) were chosen as the wavelet method due to its reliability for spectral estimations and common use in the analysis of EEG data (Mouraux and Iannetti, 2008; Pavlov et al., 2012; Ullah and Halim, 2021). Here, the EEG signal was convoluted with CMW *w*(*t*,*f*_0_) with a Gaussian shape in time (σ_*t*_) and frequency domains (σ_*f*_ = 1/2πσ_*t*_), as described in Eq. 1 (Tallon-Baudry et al., 1997):


(1) $$f:w\,\left(t,f_{0}\right)=A\,\left(e^{-t^{2}/2\,\sigma_{t}^{2}}\right)\,\left(e^{2\,i\,\pi \,f_{0}\,t}\right)$$

where *A* is the normalizing factor ${\left(1/\sigma_{t}\,\sqrt{\pi }\right)}^{1/2}$, and the wavelet family had a ratio of *f*_0_/σ_*f*_ = 6. The time-varying power *P* of the EEG signal *S*_*EGG*_ at frequency *f* was computed through the square modulus of the convolution between wavelets and *S*_*EGG*_, as shown in Eq. 2 (Tallon-Baudry et al., 1997):


(2) $$P\,\left(t,f\right)={\left|w\,\left(t,f\right)\times S_{E\,G\,G}\,\left(t\right)\right|}^{2}$$

This analysis was performed from 0 to 8 s of each trial with a resolution of 0.1 s, and from 8 to 30 Hz, with a 0.5 Hz resolution. To compute ERD/ERS, power was normalized concerning the average power calculated during the rest period (*P_R_*) in the alpha and beta frequency bands, as described in Eq. 3 (Pfurtscheller et al., 1999). Finally, grand averaged topographic brain maps were computed from the ERD/ERS data of all participants.


(3) $$E\,R\,D/E\,R\,S=\frac{P-P_{R}}{P_{R}}$$

### 2.7. Statistical analysis

A Lilliefors-corrected Kolmogorov-Smirnov test (α = 0.05) was used to determine non-Gaussian distributions for computed Sens and CA with each feedback. Thus, differences in Sens and CA with each feedback were assessed *via* a Wilcoxon signed-rank test (α = 0.05).

Statistical power of tested BCI performance differences between the continuous and the discrete feedback was performed with the G*Power analysis software (Heinrich Heine University Düsseldorf, Düsseldorf, Germany) (Faul et al., 2007). This analysis provided the probability of committing a type II statistical error with the sample of 1,440 trials for each feedback type (80 trials for each of the 18 participants included in the analysis).

ERD/ERS differences between the continuous and the discrete feedback during the MI task were assessed *via* a cluster-based permutation test. This analysis is based on non-parametric cluster randomization with multiple comparison correction (MCP) and has shown higher statistical sensitivity than traditional MCP methods such as the Bonferroni correction (Maris and Oostenveld, 2007). This method was selected after a Lilliefors-corrected Kolmogorov-Smirnov test (α = 0.05) determined that the ERD/ERS did not have a Gaussian distribution. Two separate analyses were performed for the alpha and beta frequency bands (α = 0.05). The cluster-based permutation test was implemented in MATLAB using the Fieldtrip Toolbox (Radboud University, Nijmegen, Netherlands) (Oostenveld et al., 2011). To compute the cluster analysis, the following steps were performed as proposed by Maris and Oostenveld (2007):

1. The ERD/ERS computed from each participant’s trials during MI with the continuous feedback (80 trials) were averaged, as well as, for each participant with the discrete feedback (80 trials). Then, a dataset containing the participants’ averaged ERD/ERS values for each of the 16 recorded EEG channels was created.

2. The dataset was randomly divided in two subsets with an equal number of participants’ averaged ERD/ERS values. Thus, a random partition was performed.

3. A *t*-student statistic value was computed *via* a paired *t*-student test between continuous and discrete ERD/ERS per patient.

4. Channels which ERD/ERS value was larger than the 97.5th quantile or lower than the 2.5th quantile of a *t*-distribution were selected. These selected samples were then clustered into connected sets based on spatial and temporal closeness.

5. The cluster-level statistics were computed as the sum of the *t*-values within every cluster.

6. The maximum of the cluster-level statistics was taken.

7. Steps 2–6 were repeated 5,000 times using a Monte Carlo approximation, and a histogram of the test statistics was constructed.

8. The proportion (*p*-value) of random partitions with larger cluster-level statistic values in comparison to the previously calculated histogram was computed.

9. The ERD/ERS clusters of the continuous and the discrete feedbacks are determined to be significantly different if the *p*-value is smaller than the selected critical alpha value of 0.05.

## 3. Results

**FIGURE 3:**
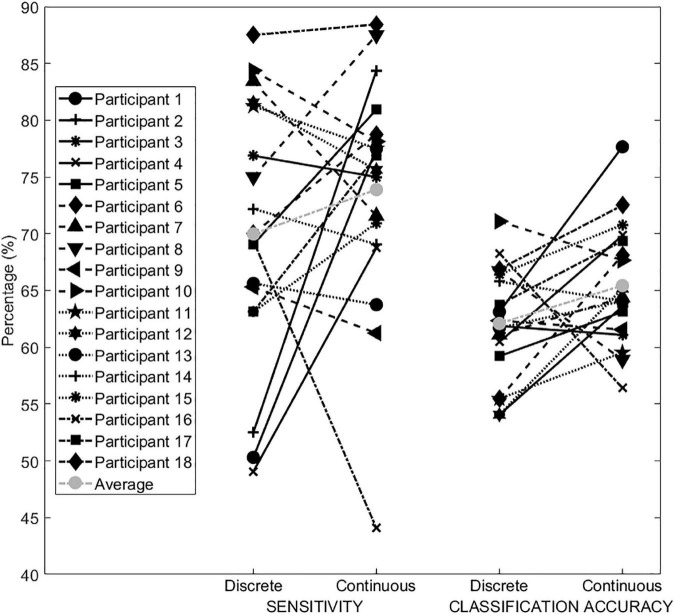
Participants’ mean Sens **(left)** and CA **(right)** achieved with the discrete and the continuous feedback.

Participants’ mean Sens and CA with the continuous and the discrete feedback during the testing phase are shown in Figure 3. Grand average Sens with the continuous feedback (mean 73.9 ± 29.1) was significantly higher (*p* = 0.001) than grand average Sens with the discrete feedback (mean 69.9 ± 28.8). Participants who achieved a higher Sens with the continuous feedback, showed greater improvements during the MI task between feedbacks (mean slope 14.9 ± 9.8) than participants who achieved a higher Sens with the discrete feedback (mean slope 7.1 ± 7.5). Similarly, grand average CA was significantly higher (*p* = 0.001) with the continuous feedback (mean 65.4 ± 17.9) in comparison to the discrete feedback (mean 62.1 ± 18.6). The participants that presented a higher CA with the continuous feedback, had a more pronounced increase in performance across feedbacks (mean slope 7.2 ± 4), compared to the participants that had a higher CA with the discrete feedback (mean slope 4.4 ± 4.5). With the continuous feedback, 12 out of 18 participants achieved a greater classification accuracy compared to the discrete feedback. Statistical power for both the Sens and the CA comparison was 0.99.

**FIGURE 4:**
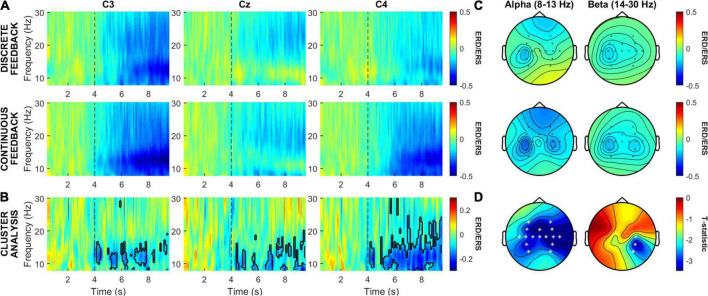
ERD/ERS analysis for the continuous and discrete feedback in the alpha and beta frequency bands. **(A)** Grand average ERD/ERS time-frequency maps of central channels C3, Cz, and C4. **(B)** Effect between the averaged ERD/ERS of the continuous and the discrete feedback in electrodes C3, Cz, and C4. **(C)** Topographic maps of the averaged ERD/ERS during the MI task in the alpha and beta frequency bands for both feedback types. **(D)** Topographical distribution of the statistically different clusters (*p* < 0.05) between the continuous and the discrete feedback.

Grand average ERD/ERS time-frequency maps for central channels C3, Cz, and C4 during MI for the continuous and the discrete feedbacks are shown in Figure 4A. These electrode locations were selected due to their importance in hand MI differentiation (Pfurtscheller et al., 2006). The effect between the time-frequency maps of both feedbacks is depicted in Figure 4B along with the statistically different clusters (*p* < 0.05) found *via* the cluster-based permutation test in electrodes C3, Cz, and C4, denoted by a black contour. Dashed lines indicate the onset of the MI task. Figure 4C shows grand average ERD/ERS topographic maps during the MI task in the alpha and beta frequency bands for both feedback types, allowing to visualize the average modulations of both bands in the complete set of recorded EEG channels. Finally, the results of the cluster-based permutation test between feedbacks’ ERD/ERS during MI, are shown in topographic maps presented in Figure 4D, where EEG channels with statistically different clusters (*p* < 0.05) between feedbacks are marked (*). In Figure 4B, more pronounced ERD for the continuous feedback is indicated in blue tones, whereas red tones denote more pronounced ERD with the discrete feedback. Figure 4D indicates a higher (blue tones) or a lower (red tones) ERD difference between feedbacks, both indicating significantly more pronounced cortical activations with the continuous feedback.

Time-frequency maps showed that with both feedbacks, cortical activations (ERD), were elicited during MI in contralateral (C3), sagittal (Cz) and ipsilateral (C4) sensorimotor regions. The cluster analysis showed statistically different clusters (*p* = 0.05) in these regions, indicating more pronounced cortical activations with the continuous feedback in alpha and beta. The largest cluster in time and frequency domains was observed in the ipsilateral region.

Topographic maps in alpha and beta showed contralateral activation in the sensorimotor cortex during MI, mainly observed in central and parietal areas, with both feedbacks. The cluster analysis showed statistically different clusters (*p* = 0.05) between both feedbacks that comprised these sensorimotor regions. In alpha, more pronounced contralateral and ipsilateral activations were observed with the continuous feedback, across all recorded cortex regions. In beta, differences were comprised by more pronounced ipsilateral activations with the continuous feedback.

## 4. Discussion

Participants’ ability to control the robotic orthosis was higher with the continuous feedback than with the discrete feedback. The averaged difference in Sens across participants was 3.9%, favoring the continuous feedback. This gain indicates that providing kinesthetic feedback during the execution of hand MI could increase the control of an EEG-based BCI system. In addition, this result is within the range of other reported changes in sensitivity between different EEG-based BCI strategies. For example, Gwak et al. (2014) reported a difference in sensitivity of 9.5% between visual and tactile feedbacks, whereas Bashashati et al. (2007) described a difference of 2.1% in sensitivity between two different 3-state asynchronous BCI systems. However, it is relevant to notice that half of the participants achieved a higher sensitivity with the continuous feedback and the other half with the discrete feedback. These results suggest that there is a possibility that a continuous kinesthetic feedback could allow certain BCI users to receive more kinesthetic stimuli during BCI control. This could be of particular relevance in the neurorehabilitation field since a higher BCI therapy dose and intensity has been associated with a better outcome in patients with physical disability, like stroke patients (Young et al., 2015). Hence, a continuous timing feedback could be advantageous for patients’ outcomes in neurorehabilitation procedures.

Participants’ average global performance with the BCI system, assessed *via* the CA, was higher with the continuous feedback compared to the discrete feedback. Although this difference in averaged performance was 3.3%, it suggested that the timing strategy of a same feedback type can have an effect on BCI control. Furthermore, this difference was higher compared to changes of less than 2% between visual feedbacks reported by Vourvopoulos et al. (2016), and also higher than 2.8% of performance difference between kinesthetic and visual feedbacks (Cantillo-Negrete et al., 2019). If individual participants’ performance is considered, more than half of the participants achieved a higher BCI control with the continuous feedback, showing the likeliness of a greater BCI control with the continuous feedback. However, this difference in control was not very different for some participants. These observations support previously supported evidence that different feedback types can have an effect in BCI control. For example, in the study by Barsotti et al. (2018) 14 out of 16 participants had a better performance with a combination of a visual and a kinesthetic vibratory feedback compared with only the visual feedback. McCreadie et al. (2013) reported that most participants performed slightly better with a visual feedback compared to an auditory feedback in the first sessions of BCI control. Furthermore, 10 out of 12 participants had a higher performance with a visual feedback comprised by the movement of a real robotic avatar, compared to a virtual feedback in the study of Batula et al. (2017). Therefore, the evidence provided by the current study implies that there is a chance of significantly improving a user’s performance with a hand MI-based BCI, if continuous kinesthetic feedback is used. Furthermore, the overall performance is competitive compared to other hand MI-based studies (Cincotti et al., 2007; Lo et al., 2016; Sollfrank et al., 2016; Barsotti et al., 2018; Zhang et al., 2020). This suggests that the choice of feedback type is important for achieving an acceptable degree of EEG-based BCI control and can be further enhanced in some participants by providing a continuous feedback strategy. Interestingly, three participants had a lower performance with the discrete feedback compared to the others, and one had a noticeable low performance with the continuous feedback. A possible explanation for these participants’ low performance with one feedback type, could be that they were part of the approximately 40% reported population that will have great difficulty in controlling a MI-based BCI, sometimes referred as BCI illiteracy (Ahn et al., 2013; Lee et al., 2019). If this is the case, then different feedback choices could be explored for these types of BCI users since it was shown that they could increase their performance with one of the feedback types that they received during the experiment. This could be important for neurorehabilitation scenarios, since patients’ withdrawal from experimental therapies comprised by BCI interventions have been attributed to frustration caused by a lack of BCI control, particularly in the first sessions of the intervention (Simon et al., 2021). Therefore, assessing if a patient can have a better performance with a continuous or a discrete feedback strategy could be potentially useful for improving patients’ chances of completing neurorehabilitation interventions.

Cortical activity was significantly more pronounced in alpha and beta bands with the continuous feedback. This difference could have been caused by a reinforcement of MI-related cortical activities due to the closer timing of MI and feedback, and a higher dosage of stimuli per trial with the continuous strategy. A lower time between task performance and stimulus could have enhanced time-dependent neuroplasticity processes, that have been related with motor learning and improvement of motor function (Murata et al., 2015). On the other hand, a direct association between the frequency of a kinesthetic stimulus and more pronounced cortical activations has been observed in animal models (Ureshi et al., 2004). In addition, movement repetition has been related to the induction of neural network changes in humans (Halder et al., 2005). Therefore, presenting more stimuli in the form of passive movement during the same time window, could have enhanced neuroplasticity mechanisms related to motor learning processes that increased cortical recruitment during MI. A relationship between more pronounced cortical activity and a continuous feedback was also reported by Shu et al. (2017) with a vibrotactile device and was attributed to an improvement of MI vividness due to the feedback. Interestingly, unlike the study of Shu et al. (2017) in the present work, stimulation was not always provided to participants during MI, but was provided only if there was a correct recognition of MI. In addition, the enhanced cortical activations were observed during MI of the dominant hand, which were not observed in the study of Shu et al. (2017) suggesting that continuous feedback triggered by MI recognition can have a higher impact on participants’ ability to elicit cortical activations.

Ipsilateral and contralateral cortical activation differences between feedbacks were observed in alpha, with ERD for the continuous feedback consistently elicited across the MI period within trials. Alpha activity has been related to motor control processes, specifically with the generation of the initial motor representation during MI (Llanos et al., 2013), and with the attentional processes required for maintaining MI (Aleksandrov and Tugin, 2012). Therefore, it can be hypothesized that the continuous feedback can aid in maintaining the level of attention required for performing MI tasks, and thus, improving the ability to perform it. This is probably achieved by providing the feedback in a shorter time window, which in turn reinforces multiple times across a single trial, the mental processes of attention and onset of the MI task. On the other hand, with beta, cortical activation differences were ipsilateral. Beta has been related with neural networks that process closed-looped sensory information during movement tasks (Athanasiou et al., 2018). Suggesting that the continuous feedback had an effect in the somatosensory cortex, but unlike the discrete feedback, this effect extended to both hemispheres by eliciting bilateral activations. Interestingly, these bilateral activations shown with the continuous feedback in both alpha and beta could be desired in neurorehabilitation scenarios, particularly those intended for stroke. This is because neuroplasticity changes in stroke often involve both hemispheres, with severely affected patients relying on the unaffected hemisphere for movement processes (Cassidy and Cramer, 2017). Therefore, feedback capable of eliciting somatosensory cortical activations among both hemispheres could enhance neuroplasticity in stroke patients.

The continuous feedback strategy could be used in BCI applications that are benefited from more feedback repetitions in a shorter time window, such as motor reinforcement learning or neurorehabilitation. In addition, most BCI applications are benefited by a higher degree of user control with the system, which seems possible for some users if a continuous feedback strategy is used. As previously mentioned, perhaps the main application of a continuous feedback strategy with MI-based BCI could be in the field of neurorehabilitation, due to the increased movement-related cortical activations that the continuous strategy provides. These enhanced activations could promote neuroplasticity in a larger degree compared to a discrete feedback strategy. For these reasons, a continuous feedback strategy could be used in a clinical trial, aimed at stroke upper extremity recovery, which would allow to clinically evaluate the effects of this feedback type in a neurorehabilitation scenario.

Finally, the current study’s limitations must be assessed. Firstly, BCI performance was heterogenous across participants and still needs to be evaluated with a larger sample in order to more precisely assess the likeliness of a higher performance with the continuous feedback. However, current results had a high statistical power (0.99) and allowed to infer that with the continuous robotic feedback some participants will achieve a significantly better performance. Secondly, effects of both feedbacks were only assessed during a short time window comprised by a single session of BCI control with each feedback. Therefore, the effects of feedback timing strategies should also be evaluated in multiple BCI control sessions, to better observe the evolution of BCI control and cortical activations elicited during MI. Despite these limitations, to the authors’ knowledge, the current study shows for the first time that a continuous feedback strategy comprised by passive movement provided by a robotic device, can have a more positive effect on EEG-based BCI control and enhanced cortical activations compared to a discrete BCI feedback strategy, suggesting its applicability in neurorehabilitation scenarios.

## 5. Conclusion

A continuous feedback strategy has the potential to increase control of a BCI based on MI decoded from EEG. Although the level of enhanced BCI control still needs to be further assessed, it is possible that some users will have a significant increase of performance with this feedback timing strategy. This enhanced BCI control can aid neurorehabilitation protocols by providing a higher performance with the system for patients, thus, helping to improve their outcomes while reducing frustration due to a lack of BCI control. In addition, the continuous feedback elicited more pronounced cortical activations in both hemispheres. Therefore, a continuous feedback strategy could be applied for neurorehabilitation, especially in stroke scenarios, where bilateral cortical activations can enhance neuroplasticity processes that improve motor function.

## Data availability statement

The datasets presented in this article are not readily available because of ethical reasons. Requests to access the datasets should be directed to the corresponding author.

## Ethics statement

The studies involving human participants were reviewed and approved by the Instituto Nacional de Rehabilitación Luis Guillermo Ibarra Ibarra Ethics Committee. The patients/participants provided their written informed consent to participate in this study.

## Author contributions

JC-N and RC-E conceived and designed the study. JC-N, RC-E, MR-G, and PC-M performed data collection. JC-N, RC-E, MR-G, and RV-C analyzed the data. RC-E and MR-G drafted and edited the manuscript. JC-N, RV-C, and PC-M provided critical revisions. All authors approved the final version of the manuscript submitted for publication.

## References

[B1] Ahn M. Cho H. Ahn S. Chan-Jun S. (2013). High theta and low alpha powers may be indicative of BCI-illiteracy in motor imagery. *PLoS One* 8:e80886. 10.1371/journal.pone.0080886 24278339 PMC3838377

[B2] Aleksandrov A. A. Tugin S. M. (2012). Changes in the Mu rhythm in different types of motor activity and on observation of movements. *Neurosci. Behav. Physiol.* 42 302–307. 10.1007/s11055-012-9566-2

[B3] Ang K. K. Chin Z. Y. Wang C. Guan C. Zhang H. (2012). Filter bank common spatial pattern algorithm on BCI competition IV datasets 2a and 2b. *Front. Neurosci.* 6:39. 10.3389/fnins.2012.00039 22479236 PMC3314883

[B4] Athanasiou A. Klados M. A. Styliadis C. Foroglou N. Polyzoidis K. Bamidis P. D. (2018). Investigating the role of Alpha and Beta rhythms in functional motor networks. *Neuroscience* 378 54–70. 10.1016/j.neuroscience.2016.05.044 27241945

[B5] Barsotti M. Leonardis D. Vanello N. Bergamasco M. Frisoli A. (2018). Effects of continuous kinaesthetic feedback based on tendon vibration on motor imagery BCI performance. *IEEE Trans. Neural Systems Rehabil. Eng.* 26 105–114. 10.1109/TNSRE.2017.2739244 28809705

[B6] Bashashati A. Ward R. K. Birch G. E. (2007). Towards development of a 3-State self-paced brain-computer interface. *Comput. Intell. Neurosci.* 2007:84386. 10.1155/2007/84386 PMC2234253 18288260

[B7] Batula A. M. Kim Y. E. Ayaz H. (2017). Virtual and actual humanoid robot control with four-class motor-imagery-based optical brain-computer interface. *Biomed. Res. Int.* 2017:1463512. 10.1155/2017/1463512 28804712 PMC5539938

[B8] Bertrand O. Perrin F. Pernier J. (1985). A theoretical justification of the average reference in topographic evoked potential studies. *Electroencephalogr. Clin. Neurophysiology/Evoked Potent. Sec.* 62 462–464. 10.1016/0168-5597(85)90058-9 2415344

[B9] Blankertz B. Tomioka R. Lemm S. Kawanabe M. Muller K. (2008). Optimizing spatial filters for robust EEG single-trial analysis. *IEEE Signal Process. Mag.* 25 41–56. 10.1109/MSP.2008.4408441

[B10] Cantillo-Negrete J. Carino-Escobar R. I. Carrillo-Mora P. Barraza-Madrigal J. A. Arias-Carrión O. (2019). Robotic orthosis compared to virtual hand for brain–computer interface feedback. *Biocybern. Biomed. Eng.* 39 263–272. 10.1016/j.bbe.2018.12.002

[B11] Cantillo-Negrete J. Carino-Escobar R. I. Carrillo-Mora P. Elias-Vinas D. Gutierrez-Martinez J. (2018). Motor imagery-based brain-computer interface coupled to a robotic hand orthosis aimed for neurorehabilitation of stroke patients. *J. Healthc. Eng.* 2018:1624637. 10.1155/2018/1624637 29849992 PMC5903326

[B12] Cantillo-Negrete J. Carino-Escobar R. I. Carrillo-Mora P. Rodriguez-Barragan M. A. Hernandez-Arenas C. Quinzaños-Fresnedo J. (2021). Brain-Computer interface coupled to a robotic hand orthosis for stroke patients’ neurorehabilitation: a crossover feasibility study. *Front. Hum. Neurosci.* 15:656975. 10.3389/fnhum.2021.656975 34163342 PMC8215105

[B13] Carino-Escobar R. I. Cantillo-Negrete J. Gutierrez-Martinez J. Vazquez R. A. (2018). Classification of motor imagery electroencephalography signals using spiking neurons with different input encoding strategies. *Neural Comput. Appl.* 30 1289–1301. 10.1007/s00521-016-2767-9

[B14] Cassidy J. M. Cramer S. C. (2017). Spontaneous and therapeutic-induced mechanisms of functional recovery after stroke. *Transl. Stroke Res.* 8 33–46. 10.1007/s12975-016-0467-5 27109642 PMC5079852

[B15] Choi J. W. Huh S. Jo S. (2020). Improving performance in motor imagery BCI-based control applications via virtually embodied feedback. *Comput. Biol. Med.* 127:104079. 10.1016/j.compbiomed.2020.104079 33126130

[B16] Cincotti F. Kauhanen L. Aloise F. Palomäki T. Caporusso N. Jylänki P. (2007). Vibrotactile feedback for brain-computer interface operation. *Comput. Intell. Neurosci.* 2007:48937. 10.1155/2007/48937 18354734 PMC2267023

[B17] Faul F. Erdfelder E. Lang A.-G. Buchner A. (2007). G*Power 3: a flexible statistical power analysis program for the social, behavioral, and biomedical sciences. *Behav. Res. Methods* 39 175–191. 10.3758/BF03193146 17695343

[B18] Fleury M. Lioi G. Barillot C. Lécuyer A. (2020). A survey on the use of haptic feedback for brain-computer interfaces and neurofeedback. *Front. Neurosci.* 14:528. 10.3389/fnins.2020.00528 32655347 PMC7325479

[B19] Gwak K. Leeb R. Millán J. del R. Kim D.-S. (2014). “Quantification and reduction of visual load during BCI operation,” in *Proceedings of the 2014 IEEE International Conference on Systems, Man, and Cybernetics (SMC)*, (San Diego, CA), 2795–2800. 10.1109/SMC.2014.6974352

[B20] Halder P. Sterr A. Brem S. Bucher K. Kollias S. Brandeis D. (2005). Electrophysiological evidence for cortical plasticity with movement repetition. *Eur. J. Neurosci.* 21 2271–2277. 10.1111/j.1460-9568.2005.04045.x 15869524

[B21] Kraeutner S. Gionfriddo A. Bardouille T. Boe S. (2014). Motor imagery-based brain activity parallels that of motor execution: evidence from magnetic source imaging of cortical oscillations. *Brain Res.* 1588 81–91. 10.1016/j.brainres.2014.09.001 25251592

[B22] Lee M.-H. Kwon O.-Y. Kim Y.-J. Kim H.-K. Lee Y.-E. Williamson J. (2019). EEG dataset and OpenBMI toolbox for three BCI paradigms: an investigation into BCI illiteracy. *GigaScience* 8:giz002. 10.1093/gigascience/giz002 30698704 PMC6501944

[B23] Leuthardt E. C. Schalk G. Roland J. Rouse A. Moran D. W. (2009). Evolution of brain-computer interfaces: going beyond classic motor physiology. *Neurosurg. Focus* 27:E4. 10.3171/2009.4.FOCUS0979 19569892 PMC2920041

[B24] Llanos C. Rodriguez M. Rodriguez-Sabate C. Morales I. Sabate M. (2013). Mu-rhythm changes during the planning of motor and motor imagery actions. *Neuropsychologia* 51 1019–1026. 10.1016/j.neuropsychologia.2013.02.008 23462240

[B25] Lo C.-C. Chien T.-Y. Chen Y.-C. Tsai S.-H. Fang W.-C. Lin B.-S. (2016). A wearable channel selection-based brain-computer interface for motor imagery detection. *Sensors (Basel)* 16:213. 10.3390/s16020213 26861347 PMC4801589

[B26] Maris E. Oostenveld R. (2007). Nonparametric statistical testing of EEG- and MEG-data. *J. Neurosci. Methods* 164 177–190. 10.1016/j.jneumeth.2007.03.024 17517438

[B27] McCreadie K. A. Coyle D. H. Prasad G. (2013). Sensorimotor learning with stereo auditory feedback for a brain–computer interface. *Med. Biol. Eng. Comput.* 51 285–293. 10.1007/s11517-012-0992-7 23197181

[B28] Monge-Pereira E. Ibañez-Pereda J. Alguacil-Diego I. M. Serrano J. I. Spottorno-Rubio M. P. Molina-Rueda F. (2017). Use of electroencephalography brain-computer interface systems as a rehabilitative approach for upper limb function after a stroke: a systematic review. *PMR* 9 918–932. 10.1016/j.pmrj.2017.04.016 28512066

[B29] Mouraux A. Iannetti G. D. (2008). Across-trial averaging of event-related EEG responses and beyond. *Magn. Reson. Imaging* 26 1041–1054. 10.1016/j.mri.2008.01.011 18479877

[B30] Murata Y. Higo N. Hayashi T. Nishimura Y. Sugiyama Y. Oishi T. (2015). Temporal plasticity involved in recovery from manual dexterity deficit after motor cortex lesion in macaque monkeys. *J. Neurosci.* 35 84–95. 10.1523/JNEUROSCI.1737-14.2015 25568105 PMC4287160

[B31] Oldfield R. C. (1971). The assessment and analysis of handedness: the Edinburgh inventory. *Neuropsychologia* 9 97–113. 10.1016/0028-3932(71)90067-4 5146491

[B32] Oostenveld R. Fries P. Maris E. Schoffelen J.-M. (2011). FieldTrip: open source software for advanced analysis of MEG, EEG, and invasive electrophysiological data. *Comput. Intell. Neurosci.* 2011:156869. 10.1155/2011/156869 21253357 PMC3021840

[B33] Pavlov A. N. Hramov A. E. Koronovskii A. A. Sitnikova E. Y. Makarov V. A. Ovchinnikov A. A. (2012). Wavelet analysis in neurodynamics. *Physics-Uspekhi* 55 845–875. 10.3367/ufne.0182.201209a.0905

[B34] Pekna M. Pekny M. Nilsson M. (2012). Modulation of neural plasticity as a basis for stroke rehabilitation. *Stroke* 43 2819–2828. 10.1161/STROKEAHA.112.654228 22923444

[B35] Pfurtscheller G. Brunner C. Schlögl A. Lopes da Silva F. H. (2006). Mu rhythm (de)synchronization and EEG single-trial classification of different motor imagery tasks. *Neuroimage* 31 153–159. 10.1016/j.neuroimage.2005.12.003 16443377

[B36] Pfurtscheller G. Lopes da Silva F. H. (1999). Event-related EEG/MEG synchronization and desynchronization: basic principles. *Clin. Neurophysiol.* 110 1842–1857. 10.1016/s1388-2457(99)00141-8 10576479

[B37] Plass-Oude Bos D. Reuderink B. van de Laar B. Gürkök H. Mühl C. Poel M. (2010). “Brain-computer interfacing and games,” in *Brain-Computer Interfaces: Applying our Minds to Human-Computer Interaction*, eds Tan D. S. Nijholt A. (London: Springer), 10.1007/978-1-84996-272-8_10

[B38] Rashid M. Sulaiman N. P. P. Abdul Majeed A. Musa R. M. Ahmad A. F. Bari B. S. (2020). Current status, challenges, and possible solutions of EEG-Based brain-computer interface: a comprehensive review. *Front. Neurorobot.* 14:25. 10.3389/fnbot.2020.00025 32581758 PMC7283463

[B39] Shi Y. Eberhart R. (1998). “A modified particle swarm optimizer,” in *Proceedings of the IEEE International Conference on Evolutionary Computatio Proceedings*, (Anchorage, AK: IEEE), 69–73. 10.1109/ICEC.1998.699146

[B40] Shu X. Yao L. Sheng X. Zhang D. Zhu X. (2017). Enhanced motor imagery-based BCI performance via tactile stimulation on unilateral hand. *Front. Hum. Neurosci.* 11:585. 10.3389/fnhum.2017.00585 PMC5717029 29249952

[B41] Simon C. Bolton D. A. E. Kennedy N. C. Soekadar S. R. Ruddy K. L. (2021). Challenges and opportunities for the future of brain-computer interface in neurorehabilitation. *Front. Neurosci.* 15:699428. 10.3389/fnins.2021.699428 34276299 PMC8282929

[B42] Sollfrank T. Ramsay A. Perdikis S. Williamson J. Murray-Smith R. Leeb R. (2016). The effect of multimodal and enriched feedback on SMR-BCI performance. *Clin. Neurophysiol.* 127 490–498. 10.1016/j.clinph.2015.06.004 26138148

[B43] Tallon-Baudry C. Bertrand O. Delpuech C. Permier J. (1997). Oscillatory gamma-band (30-70 Hz) activity induced by a visual search task in humans. *J. Neurosci.* 17 722–734. 8987794 10.1523/JNEUROSCI.17-02-00722.1997 PMC6573221

[B44] Tang Z. Li C. Wu J. Liu P. Cheng S. (2019). Classification of EEG-based single-trial motor imagery tasks using a B-CSP method for BCI. *Front. Inform. Technol. Electron. Eng.* 20:1087–1098. 10.1631/FITEE.1800083

[B45] Ullah S. Halim Z. (2021). Imagined character recognition through EEG signals using deep convolutional neural network. *Med. Biol. Eng. Comput.* 59 1167–1183. 10.1007/s11517-021-02368-0 33945075

[B46] Ureshi M. Matsuura T. Kanno I. (2004). Stimulus frequency dependence of the linear relationship between local cerebral blood flow and field potential evoked by activation of rat somatosensory cortex. *Neurosci. Res.* 48 147–153. 10.1016/j.neures.2003.10.014 14741389

[B47] Vourvopoulos A. Bermúdez I. Badia S. (2016). Motor priming in virtual reality can augment motor-imagery training efficacy in restorative brain-computer interaction: a within-subject analysis. *J. Neuroeng. Rehabil.* 13:69. 10.1186/s12984-016-0173-2 PMC4977849 27503007

[B48] Ward N. S. Cohen L. G. (2004). Mechanisms underlying recovery of motor function after stroke. *Arch. Neurol.* 61 1844–1848. 10.1001/archneur.61.12.1844 15596603 PMC3713312

[B49] Wolpaw J. R. Birbaumer N. McFarland D. J. Pfurtscheller G. Vaughan T. M. (2002). Brain–computer interfaces for communication and control. *Clin. Neurophysiol.* 113 767–791. 10.1016/S1388-2457(02)00057-3 12048038

[B50] Young B. M. Nigogosyan Z. Walton L. M. Remsik A. Song J. Nair V. A. (2015). Dose-response relationships using brain-computer interface technology impact stroke rehabilitation. *Front. Hum. Neurosci.* 9:361. 10.3389/fnhum.2015.00361 26157378 PMC4477141

[B51] Zhang D. Chen K. Jian D. Yao L. (2020). Motor imagery classification via temporal attention cues of graph embedded EEG signals. *IEEE J. Biomed. Health Inform.* 24 2570–2579. 10.1109/JBHI.2020.2967128 31976916

